# Supplementary material for: Resilience of hospital and allied infrastructure during pandemic and post pandemic periods for maternal health care of pregnant women and infants in Tamil Nadu, India ‐ A counterfactual analysis
Source: PLoS One. 2023 Sep 21;18(9):e0291749. doi: 10.1371/journal.pone.0291749 (PMC10513313; doi:10.1371/journal.pone.0291749)
Supplement: S1 File — (DOCX) [file pone.0291749.s001.docx]

**S1: Background – Selection of Method**

**Counterfactual Analysis**

A randomised controlled trial is the gold standard for assessing the effectiveness of interventions. Since the pandemic affected every area of the state in this real-world application, it is impossible for the researchers to have a control group. Using counterfactual analysis, evaluators can establish a causal relationship between interventions and results. What would have happened to beneficiaries in the absence of the intervention is examined in the "counterfactual." The difference between counterfactually predicted outcomes and those that actually occurred during the intervention is used to calculate impact.

**Model Selection**

The research revolves around the univariate time series data, which exhibits wide fluctuations and rapid changes, as one would expect from real-time data. Additionally, many temporal structures such as trend, seasonality, and holiday effect are involved. Holidays, particularly in India, are not fixed and vary from year to year. In contrast, the dates for specific holidays in Western countries are fixed according to the Gregorian calendar; the dates for many major holidays in India are determined by the Hindu calendar, which is a lunisolar calendar and thus causes holiday dates to vary each year.

While conventional forecasting methods work well and are generally reliable for limited data, high fluctuations in the data, such as in the daily CAS, makes it difficult for conventional models to provide acceptable predictions, even to the point of being unable to identify the trend. These traditional methods require highly structured data and do not work well with large volume of data [[1](#One)], as we discovered with CAS data over a six-year period. Despite the limitation of poor forecasting performance, practitioners are inclined to adopt traditional statistical techniques that are user-friendly and interpretable. Furthermore, most traditional forecasting methods present a point estimate for the prediction rather than a confidence interval. The deep learning algorithms' provision of a confidence interval allows for a range of values based on the size of the confidence interval, allowing for a more reliable forecast. Recent deep learning techniques outperform traditional machine learning algorithms for time series data [[2](#Two)]. A further benefit of using a deep learning model is the ability to predict multiple future time steps based on the number specified. Deep learning algorithms, however, are too complex and lack interpretability. They have also previously come under fire for being "black boxes" [[3](#Three)]. In order to bridge the statistical and deep learning-based approaches, the authors have adopted a scalable and interpretable model and employed a hybrid one of neural network (feedforward with AR net).

**References:**

1. Xie Y. Values and limitations of statistical models. Research in social stratification and mobility. 2011 Sep 1;29(3):343-9.
2. Nikou M, Mansourfar G, Bagherzadeh J. Stock price prediction using DEEP learning algorithm and its comparison with machine learning algorithms. Intelligent Systems in Accounting, Finance and Management. 2019 Oct;26(4):164-74.
3. Makridakis S, Spiliotis E, Assimakopoulos V. Statistical and Machine Learning forecasting methods: Concerns and ways forward. PloS one. 2018 Mar 27;13(3):e0194889.
