# Supplementary material for: Resilience of hospital and allied infrastructure during pandemic and post pandemic periods for maternal health care of pregnant women and infants in Tamil Nadu, India ‐ A counterfactual analysis
Source: PLoS One. 2023 Sep 21;18(9):e0291749. doi: 10.1371/journal.pone.0291749 (PMC10513313; doi:10.1371/journal.pone.0291749)
Supplement: S2 File — (DOCX) [file pone.0291749.s002.docx]

**S 2 Total number of ambulance active**

**Table S-2The monthly fleet strength of ambulances of Emergency Health Services of Government of Tamil Nadu during the period 2018-2022.**

| **MONTH** | **AMBULANCE COUNT** | **MONTH** | **AMBULANCE COUNT** | **MONTH** | **AMBULANCE COUNT** |
| --- | --- | --- | --- | --- | --- |
| **Apr'18** | 928 | **Nov'19** | 938 | **June'21** | 1303 |
| **May'18** | 932 | **Dec'19** | 938 | **Juy'21** | 1235 |
| **June'18** | 932 | **Jan'20** | 938 | **Aug'21** | 1235 |
| **July'18** | 936 | **Feb'20** | 938 | **Sept'21** | 1237 |
| **Aug'18** | 936 | **Mar'20** | 941 | **Oct'21** | 1237 |
| **Sept'18** | 936 | **Apr'20** | 991 | **Nov'21** | 1237 |
| **Oct'18** | 936 | **May'20** | 991 | **Dec'21** | 1237 |
| **Nov'18** | 936 | **June'20** | 1001 | **Jan'22** | 1237 |
| **Dec'18** | 936 | **July'20** | 1001 | **Feb'22** | 1237 |
| **Jan'19** | 936 | **Aug'20** | 1001 | **Mar'22** | 1353 |
| **Feb'19** | 936 | **Sept'20** | 1113 | **Apr'22** | 1353 |
| **Mar'19** | 936 | **Oct'20** | 1178 | **May'22** | 1353 |
| **Apr'19** | 936 | **Nov'20** | 1299 | **June'22** | 1353 |
| **May'19** | 936 | **Dec'20** | 1303 | **July'22** | 1353 |
| **June'19** | 936 | **Jan'21** | 1303 | **Aug'22** | 1353 |
| **July'19** | 938 | **Feb'21** | 1303 | **Sept'22** | 1353 |
| **Aug'19** | 938 | **Mar'21** | 1303 | **Oct'22** | 1353 |
| **Sept'19** | 938 | **Apr'21** | 1303 | **Nov'22** | 1353 |
| **Oct'19** | 938 | **May'21** | 1303 | **Dec'22** | 1353 |
