# Supplementary material for: Resilience of hospital and allied infrastructure during pandemic and post pandemic periods for maternal health care of pregnant women and infants in Tamil Nadu, India ‐ A counterfactual analysis
Source: PLoS One. 2023 Sep 21;18(9):e0291749. doi: 10.1371/journal.pone.0291749 (PMC10513313; doi:10.1371/journal.pone.0291749)
Supplement: S3 File — (DOCX) [file pone.0291749.s003.docx]

**S 3 Distance and Time Analysis**

The distance and duration study done for the total calls, irrespective of the type of medical emergency, slightly differs from the sub-group. For instance, both the distance travelled (measured in kilometres [km]) by the ambulance and the time consumed (measured in minutes) in respect of those calling for emergencies such as acute abdomen pain, pregnancy-related concerns, and COVID-19 illnesses were higher than the norm. On the contrary, callers who needed ambulance service for treatment as trauma victims on account of road crashes and cardio-vascular disorders had speedier transportation to the hospital and covered a lesser distance than average. Table S-2 displays those people with crises who had faster transportation and a shorter distance to go, whereas Table S-3 shows the people with emergencies who had a greater distance and a longer time.

**TABLE S-2**

| **Period** | **Distance Covered (Km)** | | | | | **Time taken to reach scene (Minutes)** | | | | | | **Number of Calls** | | | | | |
| --- | --- | --- | --- | --- | --- | --- | --- | --- | --- | --- | --- | --- | --- | --- | --- | --- | --- |
|  | **Mean** | **Median** | | **Mode** | | **Mean** | | **Median** | | **Mode** | | **Mean** | | **Median** | | **Mode** | |
| **Total Calls** |  |  | |  | |  | |  | |  | |  | |  | |  | |
| **Pre-Pandemic Period** | 8.81 | 8.81 | | 6.32 | | 21.9 | | 11.72 | | 2 | | 3338.34 | | 3275 | | 3316 | |
| **Wave 1** | 11.64 | 11.36 | | 8.72 | | 18.43 | | 12.57 | | 1.98 | | 3554.97 | | 3608 | | 2932 | |
| **Post Wave 1** | 8.12 | 8 | | 5.95 | | 13.40 | | 6.85 | | 1.98 | | 3727.53 | | 3547.5 | | 3120 | |
| **Wave 2** | 8.24 | 7.7 | | 4.93 | | 17.88 | | 6.72 | | 1.98 | | 5291.95 | | 5018 | | 4468 | |
| **Post Wave 2** | 6.99 | 7.2 | | 5.12 | | 13.34 | | 6.28 | | 1.98 | | 5679.21 | | 5133 | | 5001 | |
| **Wave 3** | 7.1 | 7.14 | | 5.29 | | 12.93 | | 6.78 | | 1.98 | | 5188.74 | | 4809 | | 4348 | |
| **Post-Pandemic Period** | 7.51 | 7.6 | | 5.72 | | 13.77 | | 8.35 | | 2 | | 5382.28 | | 4323 | | 3868 | |
| **Trauma (Vehicular)** |  |  | |  | |  | |  | |  | |  | |  | |  | |
| **Pre-Pandemic Period** | 7.26 | 7.22 | | 7.00 | | 18.44 | | 8.93 | | 1.97 | | 508.20 | | 485 | | 450 | |
| **Wave 1** | 8.58 | 8.54 | | 6.07 | | 20.80 | | 10.43 | | 1.93 | | 221.12 | | 221 | | 194 | |
| **Post Wave 1** | 6.96 | 6.92 | | 6.58 | | 10.46 | | 6.22 | | 1.98 | | 539.45 | | 516 | | 394 | |
| **Wave 2** | 7.13 | 6.98 | | 6.44 | | 15.83 | | 7.18 | | 2.00 | | 450.55 | | 485.5 | | 451 | |
| **Post Wave 2** | 6.85 | 6.82 | | 5.71 | | 12.03 | | 7.07 | | 1.83 | | 566.05 | | 544 | | 461 | |
| **Wave 3** | 6.82 | 6.84 | | 5.94 | | 12.01 | | 7.45 | | 1.98 | | 609.85 | | 578.5 | | 528 | |
| **Post Pandemic Period** | 6.81 | 6.79 | | 5.69 | | 11.50 | | 7.57 | | 2 | | 708.64 | | 618 | | 529 | |
| **Cardio/Cardiovascular** |  | |  | |  | |  | |  | |  | |  | |  | |  |
| **Pre-Pandemic Period** | 9.04 | | 9.03 | | 9.00 | | 22.26 | | 10.77 | | 1.97 | | 168.97 | | 169 | | 174 |
| **Wave 1** | 9.94 | | 9.46 | | 8.17 | | 27.83 | | 11.03 | | 1.95 | | 84.06 | | 77 | | 72 |
| **Post Wave 1** | 7.48 | | 7.50 | | 6.53 | | 11.33 | | 5.17 | | 1.82 | | 151.42 | | 147 | | 101 |
| **Wave 2** | 6.41 | | 5.96 | | 5.31 | | 13.19 | | 4.72 | | 1.88 | | 288.65 | | 200 | | 62 |
| **Post Wave 2** | 5.70 | | 5.58 | | 4.26 | | 11.52 | | 5.05 | | 1.98 | | 440.82 | | 440 | | 492 |
| **Wave 3** | 5.12 | | 4.97 | | 3.31 | | 9.88 | | 4.29 | | 1.98 | | 382.00 | | 366.5 | | 399 |
| **Post-Pandemic Period** | 7.20 | | 7.23 | | 8.00 | | 12.15 | | 6.42 | | 2.00 | | 256.60 | | 235 | | 216 |

**TABLE S-3**

| **Period** | **Distance Covered (Km)** | | | | | **Time taken to reach scene (Minutes)** | | | | | | **Number of Calls** | | | | |
| --- | --- | --- | --- | --- | --- | --- | --- | --- | --- | --- | --- | --- | --- | --- | --- | --- |
|  | **Mean** | **Median** | | **Mode** | | **Mean** | | **Median** | | **Mode** | | **Mean** | | **Median** | | **Mode** |
| **Total Calls** |  |  | |  | |  | |  | |  | |  | |  | |  |
| **Pre-Pandemic Period** | 8.81 | 8.81 | | 6.32 | | 21.9 | | 11.72 | | 2 | | 3338.34 | | 3275 | | 3316 |
| **Wave 1** | 11.64 | 11.36 | | 8.72 | | 18.43 | | 12.57 | | 1.98 | | 3554.97 | | 3608 | | 2932 |
| **Post Wave 1** | 8.12 | 8 | | 5.95 | | 13.40 | | 6.85 | | 1.98 | | 3727.53 | | 3547.5 | | 3120 |
| **Wave 2** | 8.24 | 7.7 | | 4.93 | | 17.88 | | 6.72 | | 1.98 | | 5291.95 | | 5018 | | 4468 |
| **Post Wave 2** | 6.99 | 7.2 | | 5.12 | | 13.34 | | 6.28 | | 1.98 | | 5679.21 | | 5133 | | 5001 |
| **Wave 3** | 7.1 | 7.14 | | 5.29 | | 12.93 | | 6.78 | | 1.98 | | 5188.74 | | 4809 | | 4348 |
| **Post-Pandemic Period** | 7.51 | 7.6 | | 5.72 | | 13.77 | | 8.35 | | 2 | | 5382.28 | | 4323 | | 3868 |
| **Acute Abdomen** |  |  | |  | |  | |  | |  | |  | |  | |  |
| **Pre-Pandemic Period** | 10.16 | 10.14 | | 10 | | 23.90 | | 13.15 | | 2.00 | | 261.70 | | 263 | | 270 |
| **Wave 1** | 10.90 | 10.84 | | 11.55 | | 27.90 | | 14.17 | | 1.95 | | 126.62 | | 108 | | 89 |
| **Post Wave 1** | 8.59 | 8.62 | | 8.00 | | 12.77 | | 7.55 | | 1.92 | | 188.46 | | 187 | | 106 |
| **Wave 2** | 8.05 | 7.91 | | 8.75 | | 16.54 | | 7.22 | | 1.88 | | 202.87 | | 210 | | 229 |
| **Post Wave 2** | 7.47 | 7.50 | | 5.60 | | 13.36 | | 7.45 | | 2.00 | | 310.51 | | 313.5 | | 352 |
| **Wave 3** | 7.56 | 7.64 | | 5.80 | | 13.50 | | 8.10 | | 2.00 | | 299.25 | | 297 | | 278 |
| **Post-Pandemic Period** | 8.87 | 8.85 | | 8.11 | | 14.20 | | 9.57 | | 1.93 | | 340.43 | | 315 | | 314 |
| **COVID-19** |  |  | |  | |  | |  | |  | |  | |  | |  |
| **Pre-Pandemic Period** | 0.00 | 0.00 | | 0.00 | | 0.00 | | 0.00 | | 0.00 | | 0.00 | | 0 | | 0 |
| **Wave 1** | 14.80 | 15.22 | | 7.00 | | 27.59 | | 10.10 | | 1.98 | | 1217.84 | | 1224 | | 303 |
| **Post Wave 1** | 11.79 | 11.87 | | 11.99 | | 18.42 | | 5.93 | | 2.00 | | 305.88 | | 207 | | 67 |
| **Wave 2** | 12.53 | 12.87 | | 2.80 | | 20.92 | | 6.90 | | 1.97 | | 1346.71 | | 612.5 | | 357 |
| **Post Wave 2** | 8.08 | 8.15 | | 2.92 | | 12.53 | | 3.22 | | 1.65 | | 191.52 | | 150 | | 88 |
| **Wave 3** | 8.67 | 8.91 | | 2.40 | | 15.57 | | 5.33 | | 2.00 | | 251.02 | | 145.5 | | 17 |
| **Post-Pandemic Period** | 7.94 | 6.00 | | 1.00 | | 13.21 | | 5.03 | | 1.10 | | 13.16 | | 7 | | 2 |
| **Pregnancy Related** |  |  |  | |  | |  | |  | |  | |  | |  | |
| **Pre-Pandemic Period** | 10.78 | 11.20 | 11.00 | | 25.78 | | 14.62 | | 1.98 | | 755.35 | | 656 | | 600 | |
| **Wave 1** | 11.94 | 11.93 | 7.36 | | 28.53 | | 16.50 | | 1.98 | | 735.98 | | 695 | | 634 | |
| **Post Wave 1** | 9.39 | 9.83 | 9.83 | | 14.11 | | 8.55 | | 1.98 | | 922.03 | | 741 | | 690 | |
| **Wave 2** | 8.33 | 8.24 | 3.60 | | 16.81 | | 6.73 | | 1.98 | | 1136.56 | | 774.5 | | 592 | |
| **Post Wave 2** | 7.28 | 7.77 | 3.47 | | 13.52 | | 5.90 | | 1.98 | | 1603.72 | | 1005 | | 949 | |
| **Wave 3** | 7.66 | 7.94 | 3.71 | | 13.35 | | 6.63 | | 1.98 | | 1264.31 | | 831 | | 804 | |
| **Post Pandemic Period** | 8.19 | 8.39 | 9.12 | | 14.12 | | 8.60 | | 2 | | 1532.00 | | 915 | | 792 | |
