# Supplementary material for: Resilience of hospital and allied infrastructure during pandemic and post pandemic periods for maternal health care of pregnant women and infants in Tamil Nadu, India ‐ A counterfactual analysis
Source: PLoS One. 2023 Sep 21;18(9):e0291749. doi: 10.1371/journal.pone.0291749 (PMC10513313; doi:10.1371/journal.pone.0291749)
Supplement: Attachment — Submitted filename: PLOS ONE comment REVIEW.docx [file pone.0291749.s005.docx]

Summary:

The manuscript under review provides a comprehensive exploration of the COVID-19 pandemic's influence on antenatal care (ANC) and emergency delivery services within Tamil Nadu, India. It spans over three pandemic waves from 2020 to 2022. A rigorous counterfactual analysis aided by an auto regressive neural network (AR-Net) was employed by the authors to anticipate the daily volume of ambulance service calls (CAS), which they divided into three categories. The research findings suggest that the pandemic didn't detrimentally impact emergency or scheduled prenatal care. Quite the contrary, an appreciable increase in both the number of emergency-related IFT calls and prenatal care related CAS was detected during the specified periods. This signifies a robust response from the healthcare sector.

Major Comments:

1. Relevance and Originality: This research holds significant relevance in the contemporary global landscape and expands the existing body of literature with its unique employment of counterfactual analysis and neural network modeling to assess the pandemic's impact on healthcare services.

2. Methodology: The research methodology is well-defined and aligns seamlessly with the study's objectives. The integration of AR-Net into the predictive model and subsequent data segmentation deepens the study, revealing novel applications of machine learning in the realm of healthcare data analysis.

3. Results and Conclusions: The authors have succinctly presented their findings and supported them with the collected data. The paper grants valuable insights into the resilience and adaptability of healthcare services in Tamil Nadu throughout the pandemic waves. Its conclusion advocating for the extension of ambulance services and heightened awareness carries substantial implications for healthcare policy and pandemic preparedness.

Minor Comments:

1. Writing and Structure: The manuscript maintains a high degree of professionalism and clarity in its language. The logical structure aids the reader in understanding the study's aims, methodologies, and outcomes.

2. Presentation of Findings: Although the findings are logically and clearly presented, there could be scope for enhancing the visual representation or tabulation of the data, which would facilitate easy comparison between actual and predicted results.

3. Literature Citations: While the paper draws on a satisfactory range of relevant literature, it could gain further depth by referencing studies with similar methodologies. Moreover, I would suggest considering the inclusion of Ram & Sornette's study (2021) [1] on the impact of governmental interventions on epidemic progression and workplace activity during the COVID-19 outbreak. This could provide additional support and context to the discussion.

Recommendation:

This manuscript makes a valuable contribution to understanding the global health crisis's impact on healthcare services, with a particular emphasis on maternal care. The authors' innovative approach of utilizing counterfactual analysis and AR-Net modeling warrants commendation. The implementation of the study, coupled with the presentation of its findings, is well-executed. The insights gathered carry relevance for policy-making and future research.

Hence, I recommend the paper's acceptance in its current form, albeit with minor revisions to augment data representation and further enrich the literature review.

[1] Ram, S. K., & Sornette, D. (2021). Impact of Governmental interventions on epidemic progression and workplace activity during the COVID-19 outbreak. Scientific Reports, 11(1), 21939.
